# Affinity-based electrochemical sensors for biomolecular detection in whole blood

**DOI:** 10.1007/s00216-023-04627-5

**Published:** 2023-03-14

**Authors:** Elizabeth C. Wilkirson, Kavya L. Singampalli, Jiran Li, Desh Deepak Dixit, Xue Jiang, Diego H. Gonzalez, Peter B. Lillehoj

**Affiliations:** 1 Department of Mechanical Engineering, Rice University, 6100 Main St., Houston, TX 77005 USA; 2 Department of Bioengineering, Rice University, 6500 Main St., Houston, TX 77030 USA; 3 Medical Scientist Training Program, Baylor College of Medicine, 1 Baylor Plaza, Houston, TX 77030 USA

**Keywords:** Electrochemical, Biosensor, Immunosensor, Whole blood, Diagnostics

## Abstract

The detection and/or quantification of biomarkers in blood is important for the early detection, diagnosis, and treatment of a variety of diseases and medical conditions. Among the different types of sensors for detecting molecular biomarkers, such as proteins, nucleic acids, and small-molecule drugs, affinity-based electrochemical sensors offer the advantages of high analytical sensitivity and specificity, fast detection times, simple operation, and portability. However, biomolecular detection in whole blood is challenging due to its highly complex matrix, necessitating sample purification (i.e., centrifugation), which involves the use of bulky, expensive equipment and tedious sample-handling procedures. To address these challenges, various strategies have been employed, such as purifying the blood sample directly on the sensor, employing micro-/nanoparticles to enhance the detection signal, and coating the electrode surface with blocking agents to reduce nonspecific binding, to improve the analytical performance of affinity-based electrochemical sensors without requiring sample pre-processing steps or laboratory equipment. In this article, we present an overview of affinity-based electrochemical sensor technologies that employ these strategies for biomolecular detection in whole blood.

**Graphical abstract:**

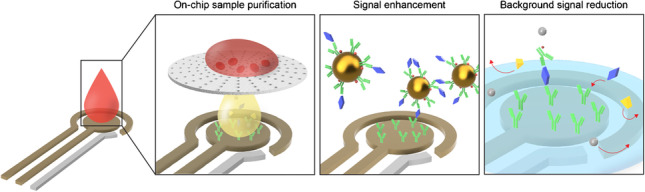

## Introduction

The detection of molecular biomarkers in bodily fluids is an instrumental aspect of clinical medicine. Specifically, the presence and/or concentration of certain biomarkers can be used to identify the risk of developing a medical condition, detect and monitor the progression of disease, or monitor therapeutic levels and response. Traditionally, the detection and/or quantification of molecular biomarkers is performed in clinical laboratories using established techniques, such as the enzyme-linked immunosorbent assay (ELISA), Western blot, or polymerase chain reaction (PCR). These methods are highly sensitive and specific due to their use of biorecognition elements that specifically bind to the target biomarker(s), but are time-consuming, require specialized equipment, and need to be performed by highly trained personnel. However, the field of medical diagnostics has seen a recent trend towards the development of simpler and faster molecular tests that offer sensitivities and specificities that are comparable, or even higher, than existing laboratory-based assays. In particular, point-of-care (POC) diagnostics can achieve accurate and rapid (< 1 h) biomolecular measurements that can be performed outside of clinical and laboratory settings. These devices typically consist of multiple components, including a biosensor, microchannels, and a detector, which are integrated into a compact platform. In addition, many POC diagnostic systems offer automated sample processing and/or fluid handling capabilities to improve usability and minimize human error [1–3]. Currently, there are several commercially available POC devices for in vitro diagnostic testing, including the Abbott i-STAT system and the Roche cobas h 232 system. While these platforms are portable, faster, and simpler than laboratory-based tests, they are expensive (US $1000’s) and have produced unreliable results for some diagnostic applications [4–6].

Electrochemical sensors are powerful analytical tools that are capable of rapid, highly sensitive biomolecular detection/quantification in biofluid samples. The compact size, minimal power requirements, low costs, and multiplexing capabilities of electrochemical sensors also make them well suited for POC testing [7]. Affinity-based sensors are a class of electrochemical sensors that utilize biorecognition elements, which are biomolecules that selectively bind to the target analyte, immobilized on an electrochemical transducer. Biorecognition elements commonly used in affinity-based sensors include antibodies, antigens, and nucleic acids (DNA, RNA, aptamers) [8, 9]. Most affinity-based electrochemical sensors employ an electroactive reporter molecule that undergoes an electrochemical reaction in response to an applied potential, resulting in the generation of an electrical signal that is proportional to the concentration of the target analyte bound to the sensing electrode [10, 11]. Compared to other biosensing modalities (e.g., optical, acoustic, piezoelectric), electrochemical sensing offers several unique advantages, such as rapid detection times, simple operation, and enhanced portability. Furthermore, electrochemical sensors can be incorporated onto disposable substrates (e.g., plastic, paper, textile), thereby reducing the cost and increasing the accessibility of this technology for global health diagnostics and POC testing [12, 13].

A major application of electrochemical sensors is the detection of biomolecules (metabolites, proteins, nucleic acids) and small-molecule drugs in blood, which is an easily accessible biofluid with a wide range of well-studied biomarkers. The molecular constituents in blood are directly associated with the physiological state of the body at any given moment, and therefore the detection/quantification of biomolecules in blood is most often used for the prevention, identification, and treatment of a variety of diseases. Specifically, the abundance (> 20,000 types) and relatively high concentrations (pg/mL to mg/mL) of proteins in blood, compared to other bodily fluids, make them ideal biomarkers for disease detection [14]. Nucleic acids are also important biomarkers in blood, as they carry the genetic information that can determine the presence of pathogens in the body or different disease states [15]. Other bodily fluids, such as saliva, urine, or sweat, contain only subsets of the biomarkers found in blood [16] typically at significantly lower concentrations [17]; therefore, these biofluids require additional processing steps for biomolecular concentration and/or signal amplification to detect these biomarkers [18]. While blood is a promising source of molecular biomarkers, sensitive biomolecular detection in whole blood is challenging since it is a complex matrix containing multiple cellular and molecular components, including blood cells, platelets, and plasma, the latter of which contains proteins (albumin, clotting factors, antibodies, enzymes, hormones), metabolites, and lipids [19–21]. Blood plasma also contains electroactive species, such as uric acid and ascorbic acid, that can cause interference effects during electrochemical measurements, resulting in a high background signal [20, 22]. The adsorption of fatty acids and irrelevant proteins on the sensing electrodes can block the target biomarker(s) from binding to the electrode surface, diminishing the detection signal and resulting in a decrease in the analytical sensitivity. The nonspecific binding of these molecules on the electrode surface can also result in a loss of specificity for the target biomarker(s) [23, 24]. Lastly, the high viscosity and presence of coagulation factors in whole blood can impede its flow through microchannels as well as hinder the transport of the target biomarker(s) and/or reporter to the sensing electrode, resulting in a low detection signal [10].

**Fig. 1:**
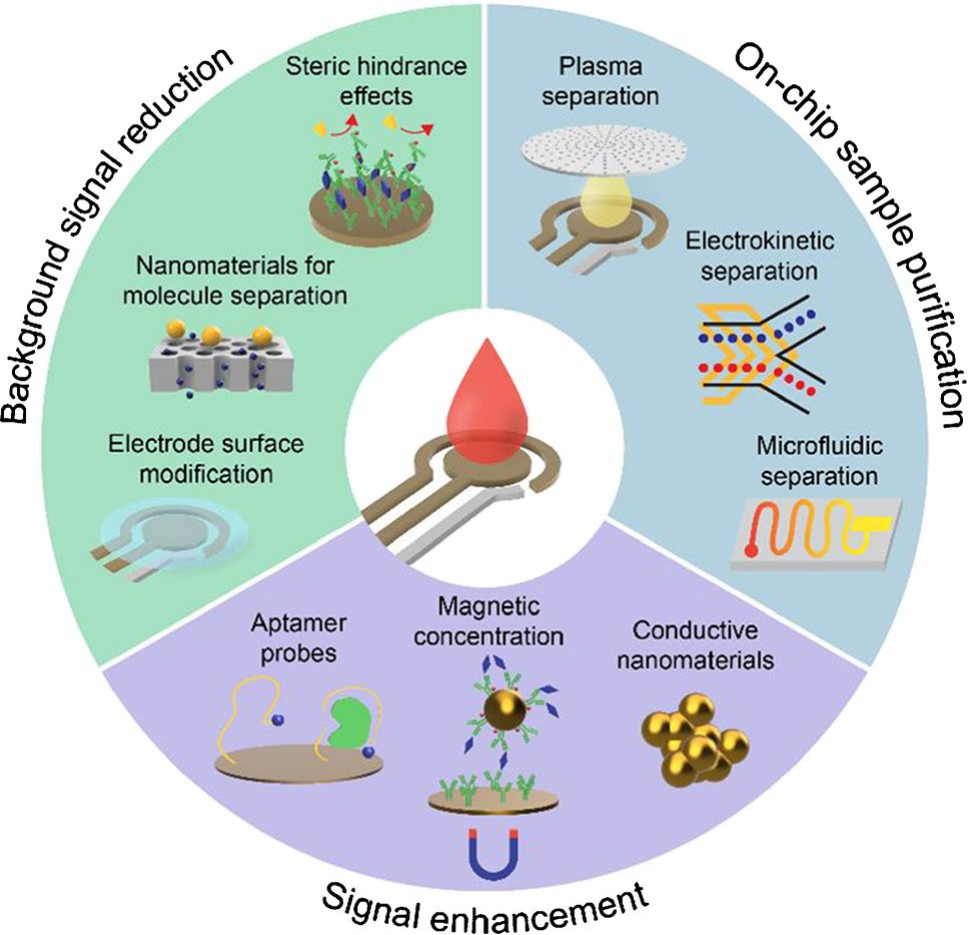
Overview of strategies to enhance the analytical performance of affinity-based electrochemical sensors for biomolecular detection in whole blood

For conventional molecular assays, whole blood is commonly processed to separate the plasma or serum to mitigate the sample matrix effects. However, this process increases the complexity of the testing protocol, hindering its use for POC testing. To address this issue, various strategies have been employed to minimize the sample matrix effects associated with whole blood and improve the performance of electrochemical sensors, in regard to their speed, analytical sensitivity, and ease of use (Fig. 1) [14]. For instance, purifying the blood sample directly on the sensor device (i.e., on chip) has shown to minimize the sample matrix effects without requiring additional pre-processing steps or laboratory equipment. Alternatively, nanoparticles have been used to increase the number of binding sites for the target biomarker(s), resulting in an amplified detection signal. Enhancement of the detection signal has also been achieved by bringing the target biomarker and/or reporter-labeled biomolecules in close proximity to the sensor surface through magnetic concentration or the use of aptamer probes. Coating the sensing electrode with blocking agents has also been shown to minimize the nonspecific binding of irrelevant proteins, thereby reducing the background signal. Additionally, utilizing steric hindrance effects to prevent electron transfer to the sensing electrodes has shown to be effective in reducing the background signal [25].

This review presents an overview of affinity-based electrochemical sensors for biomolecular detection in whole blood, focusing on strategies for improving the sensor performance (e.g., analytical sensitivity and specificity, detection time). Potential limitations, research opportunities, and future development trends in these areas are also discussed. Whole blood in this paper refers to blood that includes all of its components (e.g., blood cells, platelets, plasma) and has not been pretreated (with the exception of being diluted) prior to being loaded into the device. Technologies in which plasma separation is performed directly on the sensor in a simplified and/or autonomous manner (without requiring additional sample handling or equipment) are also included in this paper.

## On-chip sample purification

An effective method to minimize the sample matrix effects associated with whole blood is to process the sample to separate cellular components (e.g., blood cells, platelets) prior to performing the measurement. These cellular components can potentially interfere with biomolecular binding and/or electrochemical reactions and their removal from the sample can therefore increase the binding selectivity, analytical sensitivity, and repeatability. Furthermore, the removal of blood cells and platelets can improve the flow of the sample through the device and enhance biomolecular transport in the sample. Traditionally, the separation of plasma from whole blood is achieved via centrifugation, which involves the use of bulky and expensive equipment and laborious sample-handling procedures. Therefore, to simplify user operation and circumvent the need for additional instrumentation, researchers have developed sensing platforms that can process the blood sample directly on chip. These platforms can be employed outside of clinical or laboratory settings, making them ideal for POC testing [26–29]. The most common methods for on-chip sample purification that have been used for electrochemical detection in whole blood include filtration-based plasma separation, microfluidic-based plasma separation, and electrokinetic-based plasma separation.

### Filtration-based plasma separation

One of the simplest methods for separating plasma from a whole blood sample is flowing it through a membrane filter, which causes the blood cells and platelets to become trapped in the membrane, allowing the plasma to flow through to the sensing electrode. Since plasma contains nearly all of the proteins and nucleic acid biomarkers present in whole blood, it can be readily used for electrochemical sensing [30, 31]. Filtration membranes for plasma separation are commercially available and can be easily incorporated into electrochemical sensors. Due to the hydrophilic nature of these materials, the blood sample is transported through the membrane via surface tension, which circumvents the need for sample pre-treatment, pumps, or other fluidic components, making filtration-based plasma separation ideal for POC testing [32]. An electrochemical capillary-flow immunoassay employing on-chip sample purification was reported by Samper et al. [33], which utilized microfluidic circuits integrated with blood filtration membranes to detect SARS-CoV-2-specific anti-nucleocapsid (anti-N) antibodies in whole blood. A commercially available Vivid GX plasma separation membrane was used at the sample inlet of the microfluidic chip to extract plasma from the blood sample. The extracted plasma then flowed through stacked layers of polyester and double-sided adhesive films that created a capillary-flow microfluidic circuit, which allowed for reagent mixing and guidance to the detection zone containing a stencil-printed carbon electrode on top of a nitrocellulose membrane. This device was able to detect SARS-CoV-2 anti-N antibodies in spiked whole blood at concentrations as low as 5 ng/mL via chronoamperometry. The Vivid plasma separation membrane was also used by Kikkeri et al. [31] in an electrochemical immunosensor for the detection of interlukin-6 (IL-6). Processing the blood sample through the plasma separation membrane resulted in > 99% separation efficiency for the removal of blood cells. After filtration, anti-IL-6-functionalized magnetic beads (MBs) were used to capture the target IL-6 proteins to enhance the analytical sensitivity of the sensor. Measurements of IL-6 were performed in spiked 3×-diluted whole blood samples, which revealed that this sensor could detect IL-6 at concentrations as low as 40 pg/mL with < 15% deviation from the theoretical concentrations.

**Fig. 2:**
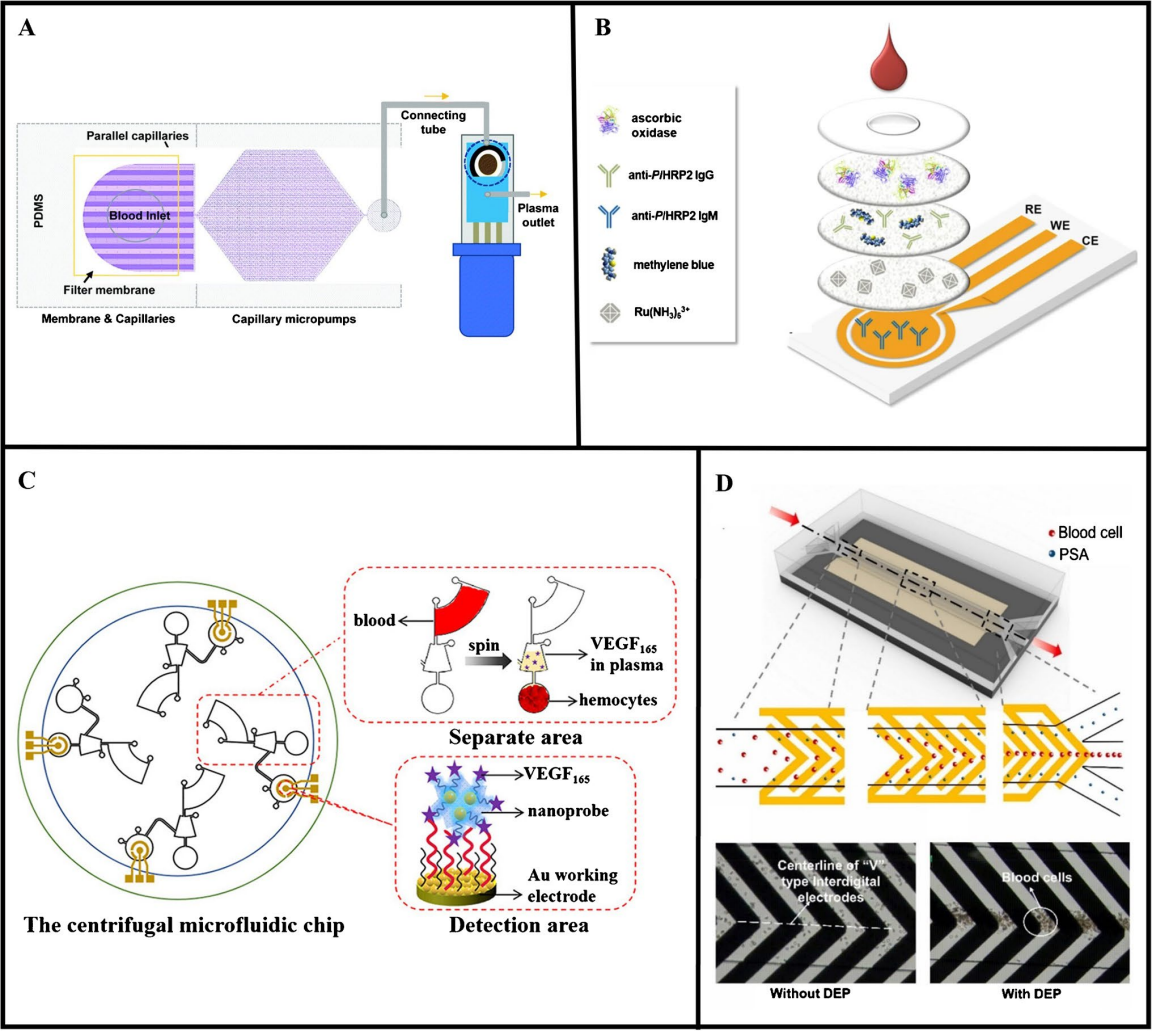
Strategies for on-chip plasma separation. **A** Separation of blood cells from a whole blood sample via filtration through a membrane connected to a series of microfluidic capillaries and capillary micropumps. Used with permission of Royal Society of Chemistry, from [34]; permission conveyed through Copyright Clearance Center, Inc. **B** Filtration of a whole blood sample through a stacked membrane assembly for the quantification of *Pf*HRP2. Reprinted from [22]. **C** Centrifuge-induced flow through microchannels and collection chambers for automated plasma separation and electrochemical detection on a lab-on-a-disc platform. Reprinted (adapted) with permission from [39]. Copyright 2022 American Chemical Society. **D** DEP-induced separation of blood cells from a whole blood sample inside a microfluidic device. Reprinted from [41] with permission from Elsevier

While processing whole blood samples using a filtration membrane is simple and effective, the amount of plasma that can be extracted is relatively low (~ 1–5 µL). To obtain larger volumes of plasma using filtration, Keyvani et al. [34] developed an affinity-based electrochemical sensing platform that employed a membrane embedded in a microfluidic chip with a network of parallel capillaries and micropumps, as shown in Fig. 2A. The integration of the filtration membrane with the capillaries and micropumps allowed for the autonomous collection and continuous extraction of plasma from whole blood, resulting in the separation of up to 22 μL of plasma from 160 μL of whole blood. This platform was used for the detection of high-risk human papilloma virus (hr-HPV16) DNA, which could be detected at concentrations as low as 0.48 μM (~ 10E + 9 copies/mL) in spiked whole blood in approximately 20 min.

On-chip plasma separation has also been combined with other sensor enhancement strategies to amplify the detection signal. Dutta and Lillehoj [22] created a stacked membrane assembly that was combined with an electrochemical immunosensor for wash-free and label-free detection of *Plasmodium falciparum* histidine-rich protein 2 (*Pf*HRP2). The stacked membrane assembly was situated above a gold (Au) electrochemical sensor that was coated with capture anti-*Pf*HRP2 IgM antibodies, and was comprised of a Vivid plasma separation membrane and two cellulose membranes, which contained dried protein and redox reagents, including a detection antibody (anti-*Pf*HRP2 IgG), methylene blue, and Ru(NH_3_)_6_^3+^ (Fig. 2B). To initiate the measurement, a drop of whole blood was dispensed onto the membrane assembly. As the blood flowed through the plasma separation membrane, the blood cells and platelets were filtered out, allowing the plasma to flow through to the underlying cellulose layers. The dried reagents were reconstituted as the plasma flowed through the cellulose layers and the plasma sample was subsequently transported to the electrochemical sensor via capillary flow. A unique detection scheme based on electrochemical-chemical redox cycling was employed for signal amplification, which was combined with an affinity-based sensing scheme where surface attachment of the target *Pf*HRP2 antigen formed an insulator layer on the sensor surface, impeding electron transfer and diminishing the electrochemical signal. Proof-of-concept studies were performed by using this immunosensor for quantitative measurements of *Pf*HRP2, which could be detected in spiked whole blood samples from 100 ng/mL to 100 μg/mL within 5 min.

### Microfluidic-based plasma separation

Microfluidics offers several advantages, including enhanced automation, faster processing times, and reduced sample waste, over conventional benchtop methods for sample processing [35]. The small dimensions of microchannels also allow for the separation of cellular components and larger biomolecules from plasma, which can be subsequently transported to the sensing electrodes. This method was employed by Lim et al. [36] for the development of a microfluidic electrochemical immunoassay for the detection of histamine in whole blood. This device consisted of a multi-channel matrix column coated with cation-exchange functional groups for increased surface area for optimized cation exchange. The matrix column captured and separated irrelevant antigen–antibody complexes and unreacted antibodies, allowing only the target histamine-antibody complex to flow through the microchannels to the sensing electrodes. This device was able to detect histamine in a 10 μL blood sample at concentrations from 200 to 2000 ng/mL within 2 min. In addition to offering rapid and automated filtration of whole blood, the microchannel matrix column was able to process small sample volumes, which is difficult to achieve using conventional filtration methods. In an alternative approach, Fan et al. [37] reported a microfluidic chip for protein analysis in small blood volumes using the Zweifach-Fung effect. The Zweifach-Fung effect combines higher-flow primary microchannels with lower-flow supplementary microchannels that branch off at precise angles to naturally coerce the separation of blood cells from whole blood. Due to the flow resistance ratio between the primary and supplementary microchannels, a streamline was formed close to the primary channel wall. Blood cells with a radius larger than the distance between the streamline and the primary channel wall were directed to waste outlets and the resulting plasma was directed into the supplementary channels. The plasma then flowed into a barcode-like, high-density microfluidic network that contained a microarray coated with capture antibodies via DNA-directed immobilization, enabling the quantitative detection of multiple target proteins, including prostate-specific antigen (PSA), complement C3, C-reactive protein (CRP), and plasminogen, in a few hundred nanoliters of plasma from a finger prick blood sample.

Microfluidic devices combining microchannels and microfluidic components with electrochemical sensors have also been developed as “all-in-one” platforms for integrated sample processing and biomolecular detection. This approach has been used for the development of lab-on-a-disc or lab-on-a-CD platforms where centrifugal force drives the flow of fluids through microchannels embedded within a plastic circular disc [38]. He et al. [39] developed a centrifugal microfluidic immunosensor that contained an embedded blood separation unit consisting of a series of microchannels and small collection chambers, for separating plasma from whole blood. To initiate the measurement, 50 μL of whole blood and 40 μL of PBS/nanoprobe solution (for plasma dilution) were dispensed into corresponding inlets, followed by spinning the disc at 3000 rpm for 4 min. As shown in Fig. 2C, the centrifugal force caused the blood sample to flow from the blood chamber to the plasma chamber, during which the blood cells were separated and retained in a different chamber. The connecting microchannel prevented the plasma from remixing with the blood cells. The purified plasma sample then flowed through a curved microchannel into a detection unit consisting of a working electrode coated with synthetic nanoprobes that were designed to bind to vascular endothelial growth factor 165 (VEGF_165_). Measurements of VEGF_165_ in whole blood revealed that this centrifugal microfluidic immunosensor exhibited a lower limit of detection (LOD) of 0.67 pg/mL.

### Electrokinetic-based plasma separation

Electrokinetics, which leverages the interaction between applied electrical field forces and fluidic forces (i.e., drag), has also been used to separate out cellular components from whole blood. Unlike plasma separation techniques based on centrifugal forces, which are influenced by machine specifications, electrokinetics is a nonmechanical technique that only involves the application of an external electric field, and therefore, the operations are less variable and the results are more reliable. In addition, electrokinetic-based separation techniques can be readily integrated with microfluidic devices for precise manipulation of biological particles and fluids [40]. One such example was reported by Wang et al. [41] who developed a microfluidic affinity-based electrochemical sensing platform utilizing dielectrophoresis for plasma separation for the detection of PSA. In this device, the dielectrophoretic force and fluid drag force created a streamline effect in the center of the microchannel, causing the blood cells to migrate towards the outlet. The plasma remained along the walls of the microchannel and flowed into separate smaller channels (Fig. 2D), resulting in a separation efficiency of 98%. The plasma then flowed through a series of serpentine channels for mixing with electrochemical nanoprobes comprised of bovine serum albumin (BSA), detection aptamer 2, polythionine, and gold nanoparticles (AuNPs). PSA in the plasma bonded to the nanoprobes resulting in the formation of PSA-nanoprobe conjugates, which were used to generate an electrochemical signal. Using this platform, PSA could be detected at concentrations as low as 0.25 pg/mL in whole blood.

Each of the strategies discussed in this section demonstrates the feasibility of separating plasma from whole blood directly on chip, thereby minimizing the sample matrix effects and enhancing the sensor performance. In each of the presented technologies, sample processing was automated, which reduced the overall detection time and simplified the testing protocol. However, there are several limitations associated with these strategies, such as the need for larger blood volumes since plasma constitutes a small volume within the blood, the complexity (and cost) of fabricating precise microfluidic components, and the use of additional hardware (e.g., pumps, spindle motors, power supplies), all of which hinder their usefulness for POC testing. Therefore, alternative strategies to improve the performance of electrochemical sensors for biomolecular detection in whole blood samples without plasma separation, such as amplifying the detection signal or reducing the background signal, have been demonstrated and will be discussed in the following sections.

## Signal enhancement

A commonly used approach to increase the analytical sensitivity of electrochemical sensors is to amplify the detection signal. This can be achieved through various means, such as increasing the amount of the target biomarker that binds to the sensing electrode, increasing the amount of the reporter involved in the electrochemical reaction, or enhancing the transfer of electrons between the reporter and electrode surface. All of these can be achieved by incorporating nanomaterials with electrochemical sensors. This strategy leverages the large surface area-to-volume ratio of nanomaterials to provide more binding sites for the target biomarker and/or reporter, resulting in an amplified detection signal. The use of conductive nanomaterials enhances the electrical properties of the electrodes, which has shown to promote electron transfer between enzymatic reporters and sensing electrodes, further amplifying the detection signal and improving the analytical sensitivity [42, 43]. Furthermore, coating the sensing electrode with carbon-based nanomaterials with high electron mobility (e.g., graphene) can also amplify the detection signal [44, 45].

Micro- and nano-sized magnetic beads (MBs) have also been utilized in electrochemical sensors to improve the sensor performance. In addition to offering a larger surface area, biomolecule-labeled MBs can be functionalized for the efficient capture of the target biomarker and controlled for optimized mixing patterns [46, 47]. Additionally, MBs can be quickly transported within the sample and concentrated onto the electrode surface using an external magnet, which reduces the detection time and facilitates electron transfer during electrochemical reactions. Similarly, aptamer probes, which can change in conformation when bound to the target biomarker, offer improved electron transfer by bringing the reporter close to the electrode surface [48]. In the following section, we present several strategies to enhance the electrochemical signal for biomolecular detection in whole blood. We also discuss how these individual strategies can be combined to further enhance the sensor performance.

### Conductive nanomaterials

AuNPs are among the most commonly used nanomaterials for modifying electrochemical sensor electrodes due to their high conductivity, ease of functionalization, and excellent biocompatibility. Most notably, the attachment of AuNPs to sensing electrodes effectively increases their surface area and provides more binding sites for the target biomarker and/or reporter, which enhances the detection signal [49]. Liu et al. [50, 51] developed a label-free electrochemical impedance immunosensor and an amperometric sensor with AuNP/oligo(ethylene glycol) (OEG-COOH)-modified electrodes for the detection of hemoglobin A1c (HbA1c). The AuNPs were modified with N-glycosylated pentapeptide, which exhibited a high affinity to bind with anti-HbA1c IgG, while the use of OEG-COOH prevented nonspecific binding on the sensor surface. Experiments evaluating the electrical properties of the sensors revealed a small change in charge transfer resistance due to the AuNP/OEG-COOH electrode modification. HbA1c measurements were performed in a human whole blood sample, resulting in the detection of 4.88% ± 1.04% HbA1c by the impedance sensor and 5.11% ± 0.09% HbA1c by the amperometric sensor, both of which were comparable to clinical laboratory results.

In addition to nanospheres, nanoparticles with other geometries, such as nanorods and nanowires, have been used to increase the surface area for increased analyte binding and electron transfer. For example, Dong et al. [52] employed Au nanocones that were electrochemically deposited on a carbon fiber electrode for the detection of hydrogen peroxide (H_2_O_2_) in blood. A novel recognition molecule, 5-(1,2-dithiolan-3-yl)-N-(4-(4,4,5,5-tetramethyl-1,3,2-dioxaborolan-2-yl)phenyl)pent-anamide (BA), was designed, synthesized, and bound to the nanocones, which reacted with H_2_O_2_ to generate an electrical current. The use of the nanocones resulted in an increase in the charge transfer from the electrode and allowed a larger amount of BA to be immobilized on the surface, enabling sensitive detection of H_2_O_2_ at concentrations from 0.5 to 400 μM with a lower LOD of 0.02 μM. Mahshid and Dabdoub [53] deposited high-curvature Au nanostructures on glass chips and employed them as working electrodes to develop an electrochemical sensor for the detection of otolin-1 and prestin. The Au nanostructures resembled tree-like formations, which ensured a high surface density for the immobilization of DNA capture probes. Using this sensor, measurements of otolin-1 were performed in spiked whole blood, which exhibited a lower LOD of 2 pM and each measurement could be completed within 10 min.

Carbon-based nanomaterials have also been used for signal amplification in electrochemical sensors due to their high electrical conductivity, excellent stability, and biocompatibility [44]. These nanomaterials are available in a variety of geometries, such as nanotubes, nanofibers, and nanoplatelets, each having different physical, electrical, and mechanical properties which can be used to modulate the sensor performance [49]. Carbon nanotubes (CNTs) and graphene nanoparticles have been extensively used to modify electrochemical sensors to increase the sensing electrode surface area and enhance the electrical properties, biocompatibility, and stability of the sensor [54]. For example, Thiruppathi et al. [55] investigated the use of various carbon-based nanomaterials, including CNTs, hollow carbon nanoparticles, and graphene nanoribbons, for the development of a screen-printed carbon electrode electrochemical sensor for the detection of total hemoglobin (tHb) in whole blood. Multiwall CNTs with Nafion (CNT-Nf) were selected for modifying the electrodes over hollow carbon nanoparticles or graphene nanoribbons since they offered the largest signal amplification. Using cyclic voltammetry, the CNT-Nf modified sensor produced a well-defined redox peak that correlated closely with tHb in standard samples, validating the accuracy and stability of the modified sensor. Square wave voltammetry was then used for simultaneous measurements of HbA1c and tHb, which were used to calculate the HbA1c %. This sensor exhibited a lower LOD of 4.2 nM when detecting HbA1c and a coefficient of variation of 3.37%, while requiring only 2 μL of whole blood for each measurement.

### Magnetic concentration

MBs are widely used in molecular assays and biosensors due to their ability to be easily manipulated and concentrated using an external magnet. For example, MBs functionalized with biorecognition elements that selectively bind to a target biomarker enable rapid biomolecular isolation/purification from biofluid samples. Similar to the use of conductive nanomaterials as discussed above, affinity-based sensors benefit greatly from the inclusion of functionalized MBs due to the increased surface area, which allows for the attachment of more target biomarkers and/or reporter molecules on the sensing electrode [56]. Magnets can also facilitate the transport and mixing of functionalized MBs in the sample, which allows for more interactions and subsequent binding with the target biomarker. Most importantly, the use of MBs enables the rapid magnetic concentration of MB-target conjugates on the sensing electrode, which facilitates electron transfer during electrochemical reactions, leading to an increase in the detection signal.

**Fig. 3:**
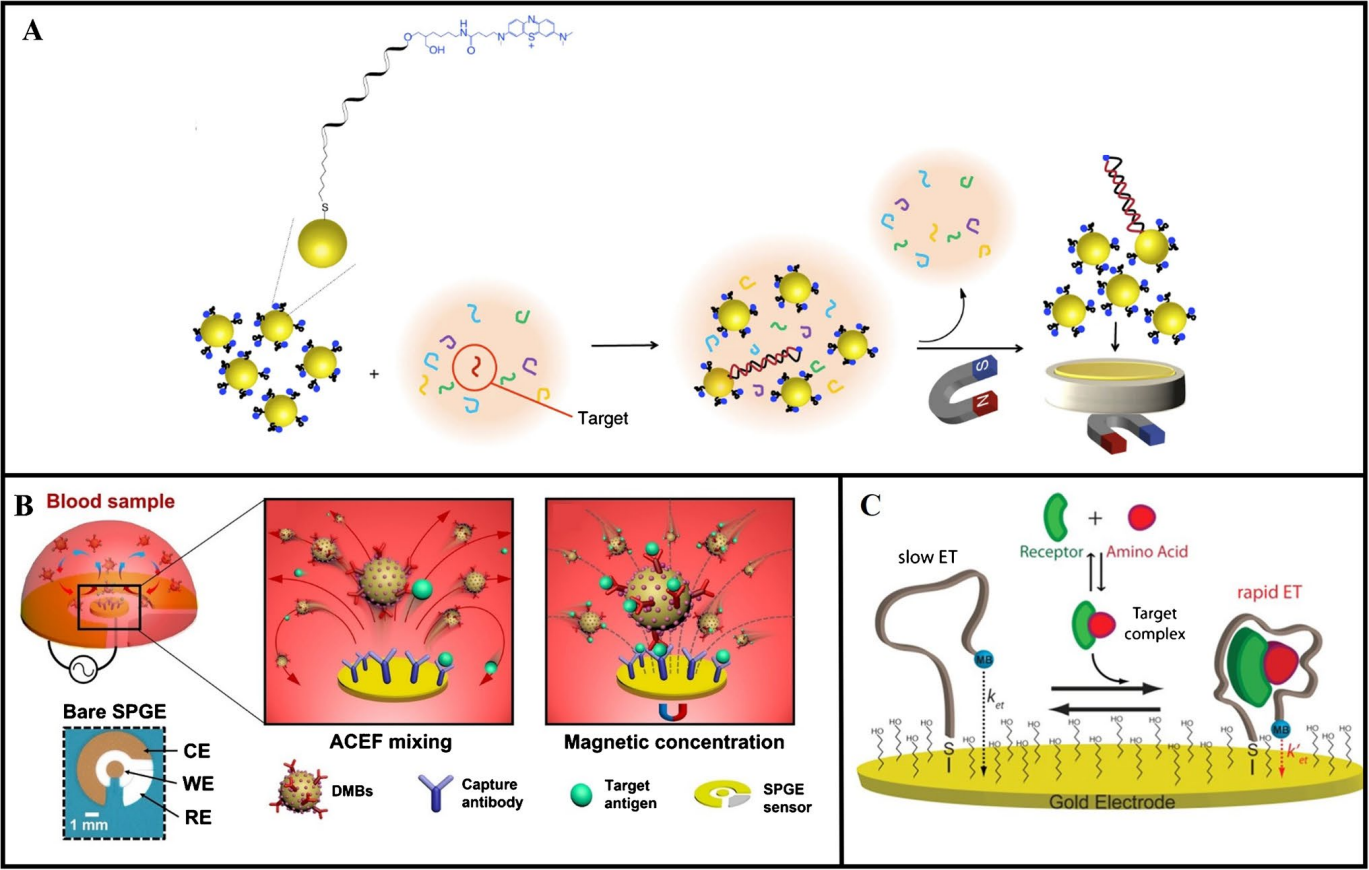
Strategies for amplifying the detection signal. **A** Magnetic separation of MB-target conjugates from a blood sample and subsequent concentration on the working electrode. Reprinted (adapted) with permission from [57]. Copyright 2021 American Chemical Society. **B** Enhanced mass transport of MB-target conjugates in blood using AC electrothermal flow mixing. Reprinted from [63]. **C** Enhanced electron transfer from the reporter to the sensing electrode resulting from aptamer-based conformational change following binding with the target complex. Reprinted (adapted) with permission from [66]. Copyright 2019 American Chemical Society

The immobilization of functionalized MBs on the sensing electrode has also been used to encourage binding and enhance electron transfer for signal amplification. This approach was demonstrated by Tavallaie et al. [57] for the detection of microRNA-21 (miR-21) in whole blood using a network of Au-coated MBs modified with a redox-labeled probe DNA sequence (DNA-Au@MNPs) (Fig. 3A). Briefly, DNA-Au@MNPs were combined with the whole blood sample and incubated for 30 min. A magnet was used to separate the miR-21-DNA-Au@MNP conjugates from the sample, which were subsequently concentrated on the surface of an Au microelectrode by placing the sensor on another magnet. After 5 min, 10 cycles of square wave voltammetry were applied, then the peak currents were used to measure the amount of miR-21 present in the sample. Using this method, the sensor was able to detect miR-21 in whole blood at concentrations from 10 aM to 1 nM. This approach has also been used for the detection of protein biomarkers, as demonstrated by Molinero-Fernández et al. [58], who developed an electrochemical magneto-immunosensor for the detection of CRP in 10×-diluted whole blood. In this work, the blood sample was first incubated for 15 min with a biotinylated capture antibody bound to streptavidin-functionalized MBs and a detection antibody labeled with HRP. A magnet was placed underneath the sensor for 5 min, which concentrated the MB-CRP immunocomplexes on the working electrode, followed by electrochemical detection via amperometry. Using this immunosensor, CRP could be detected at concentrations as low as 1.5 ng/mL in < 15 min. Similarly, Liang et al. [59] employed a magnetic immobilization method to control the location of avidin-functionalized MBs on the working electrode of an electrochemical immunosensor after 18 min of incubation with a Prussian blue core–shell nanomaterial labeled with detection antibodies. This sensor was used to detect N-terminal (NT)-pro hormone BNP (NT-proBNP), which could be detected at concentrations as low as 3 pg/mL in whole blood.

The use of an external magnetic field to concentrate and localize functionalized MBs has also been used in a paper-based electrochemical sensor. Ruiz-Vega et al. [60] demonstrated an electrochemical immunosensor for the rapid detection of *Plasmodium falciparum* lactate dehydrogenase (*Pf*LDH) in whole blood samples using MBs. This device consisted of a single-use, paper-based microfluidic device integrated with double-sided screen-printed carbon electrodes for electrochemical detection. As the sample flowed through the paper microchannel, the target *Pf*LDH protein bound to capture antibody-coated MBs and poly-HRP-conjugated detection antibodies before reaching the electrode surface. An external magnet was used to concentrate the MB-*Pf*LDH immunocomplexes on the working electrode followed by amperometric measurements. This paper-based immunosensor was able to detect *Pf*LDH in 25×-diluted lysed blood at concentrations as low as 8.5 ng/mL in < 20 min.

While the use of an external magnetic field is a simple and efficient method to control the transport of MBs, several research groups have demonstrated other strategies to encourage the transport and mixing of MBs with other reagents, combined with magnetic concentration, to further enhance the analytical sensitivity. A unique strategy for enhancing the mass transport of MBs in blood to facilitate binding between MBs and the target biomarker was demonstrated by Fang et al. [61]. In this study, MBs labeled with Prussian blue and iron (II, III) oxide, modified with oxidized low-density lipoprotein (Ox-LDL) antibody and BSA, and bound to magnesium microspheres, were dispersed in whole blood. Due to the magnesium microsphere’s interaction with the water in the blood, hydrogen gas was generated, which created a driving force for the mass transport of the microparticles, termed “micromotors.” The micromotors captured Ox-LDL in the blood for 90 min, then the Ox-LDL-MB conjugates were magnetically concentrated on a magnetic glassy carbon electrode using a funnel. The electrode was modified with multi-walled CNTs to improve the electrical conductivity of the sensor, which exhibited a lower LOD of 9.80 × 10^−4^ μg/mL in whole blood. Using a similar approach, Wang et al. [62] employed “microswimmers” to detect miRNA and thrombin in whole blood. The microswimmers were magnesium-based microspheres modified with a magnetic nanocomposite of magnesium, ferric oxide, polydopamine, and heparin, and functioned in the same manner as the previously mentioned micromotors when placed in whole blood. The microswimmers interacted with trapping agents composed of magnesium ion-induced nucleic acid fragments specific to the target biomarkers. Using this technique, concentrations as low as 4.73 aM for miRNA and 29.60 aM for thrombin could be detected in whole blood samples.

Li and Lillehoj [63] combined AC electrothermal flow (ACEF) with a magneto electrochemical immunosensor for the detection of *Pf*HRP2 in 5×-diluted whole blood (Fig. 3B). MBs dually labeled with HRP-conjugated anti-*Pf*HRP2 detection antibodies and free HRP were mixed with the blood sample and incubated on a screen-printed Au electrode sensor coated with anti-*Pf*HRP2 capture antibodies. An AC electric field was applied to the sensor electrodes, resulting in nonuniform Joule heating, which generated microflows in the sample. The ACEF-induced flows accelerated the transport of MBs in the blood sample, facilitating the formation of immunocomplexes with *Pf*HRP2. After 4 min of ACEF mixing, a magnet was placed under the sensor for 1 min causing the MB-immunocomplexes to concentrate on the working electrode. Using this approach, *Pf*HRP2 could be detected at concentrations as low as 5.7 pg/mL in 7 min, which is 20× faster than conventional ELISA protocols.

### Aptamer probes

The detection signal generated by an electrochemical sensor can be increased by reducing the distance that the electrons need to flow between the reporter and the working electrode. One approach to achieve this has been the use of shape-changing aptamers as biorecognition elements. Aptamers have been used as recognition elements for the detection of various types of analytes including ions, nucleic acids, small molecules, and proteins, and offer several advantages over antibodies, such as ease of synthesis, higher stability, increased specificity, and tunable affinity [64]. In this approach, the aptamer is labeled with a reporter molecule and immobilized on the working electrode. When the target biomarker binds to the aptamer, it undergoes a conformational change causing the reporter to be brought closer to the electrode surface, facilitating electron transfer during the electrochemical reaction, thereby enhancing the detection signal [65]. Idili et al. [66] developed an aptamer-based electrochemical sensor for the detection of phenylalanine in 1000×-diluted blood. The aptamer underwent a conformational change when bound to a phenylalanine-rhodamine complex, enabling the detection of phenylalanine at concentrations between 0.1 and 10 µM within 10 min. By using an aptamer with a more open initial conformation, a significant shape change between the bound and unbound states of the aptamer was obtained (Fig. 3C), resulting in a large signal gain as well as increased sensitivity. A similar method using aptamers labeled with a methylene blue reporter was employed to detect tumor necrosis factor-alpha (TNF-α) with a lower LOD of 58 pM in whole human blood [67] and VEGF with a lower  LOD of 5 pM in 50% blood serum [68].

Aptamers can also be modified to bind a wider range of molecules by modifying the unbound conformation state of the molecule. This approach was utilized by Kurnik et al. [69] to develop an aptamer-based electrochemical sensor that discriminates between a protein’s peptide targets in whole blood for continuous, real-time sensing. In this work, the SH3 domain from human Fyn kinase (FynSH3) was destabilized to open up the protein binding domains, enabling the binding of multiple molecules, and modified with a methylene blue reporter to generate a detection signal. When bound to the high-affinity peptide ligand VSL12, the target biomarker, the change in protein conformation brought the reporter closer to the working electrode, producing a signal gain proportional to the binding affinity of the target. This sensor exhibited high specificity to the target receptor, achieving a lower LOD of 2.5 μM in bovine blood, which is 30× lower than the protein’s dissociation constant.

Aptamer-based sensors also offer enhanced stability for long-term or real-time measurements in whole blood. Towards this end, Li et al. [70] developed an aptamer-based electrochemical sensor for the detection of small drug molecules in undiluted bovine blood. Measurements were performed using square wave voltammetry to continuously monitor changes in electron transfer kinetics over time by utilizing a “dual-frequency” approach. By comparing the detection signal produced between the bound and unbound states, cocaine and doxorubicin were detected in a calibration-free method with a measurement accuracy within 20% of the spiked target concentration across a 20- to 100-fold range, which is comparable to commercial sensors that are accurate within 20% across a 30-fold concentration range. Tetrahedral DNA nanostructures (TDNs) were used by Li et al. [71] to create an aptamer-based electrochemical sensor for rapid, one-step, real-time detection of DNA in whole blood. This sensor utilized TDNs with two functional strands that undergo binding-induced conformational change on the electrode surface, producing a change in the electrical current via square wave voltammetry. The authors claim a lower LOD of 300 fM for DNA detection, although whether this was obtained in serum or whole blood was unspecified. Additionally, this sensor exhibited minimal (< 11%) current drift when placed in whole blood for up to 10 h, suggesting that it could be used for continuous real-time analysis in whole blood.

While the use of aptamer probes in electrochemical sensors has shown to enhance the detection signal, the high ionic strength of whole blood can hinder the detection of some proteins due to the Debye shielding effect [72]. To overcome this issue, other signal enhancement strategies, such as the use of nanomaterials, can be combined with aptamer probes to fine-tune the surface properties of the sensing electrode to minimize the Debye shielding effect, resulting in more efficient electron transfer and amplification of the detection signal [45]. Eissa and Zourob [73] employed this technique to develop an AuNP-enhanced, aptamer-based electrochemical biosensing platform for the detection of HbA1c and tHb in whole blood. This sensor was comprised of carbon electrodes decorated with AuNPs, which were subsequently coated with thiolated aptamers. Square wave voltammetry measurements were performed using buffer samples spiked with HbA1c and tHb, which resulted in lower LODs of 0.2 ng/mL and 0.34 ng/mL, respectively. Measurements of HbA1c % were also performed in 100–100,000×-diluted whole blood and were calculated to be 6.67–10.47%, which were in good agreement with clinically established results determined photometrically by measuring the ratio of the HbA1c peak area over the tHb peak area.

Li et al. [74] demonstrated an alternative signal amplification method in combination with aptamers based on a hybridization chain reaction (HCR) that employed “One reporter” and “N reporters,” which worked together to form an “O-N” approach to detect nucleic acids. Two redox reporters (ferrocene and methylene blue) were attached to specific sites of the HCR-activated DNA strands. Upon binding to the target molecule, the capture DNA changed conformation, enabling it to bind to additional strands of DNA containing reporter molecules, thus amplifying the detection signal. Additionally, the reporters generated current peaks at distinguishable potentials, with minimal (< 4-fold variations among one another) sensor-to-sensor variations in the detection signal, circumventing the need for calibration. Through the optimization of the reporters on the DNA strands, this sensor was able to detect circulating tumor DNA (ctDNA) at concentrations from 0.1 pM to 1 nM in undiluted whole blood.

## Background signal reduction

A major challenge with achieving sensitive biomolecular detection in whole blood is the adsorption and/or nonspecific binding of irrelevant proteins on the electrode surface, which blocks the target biomarker from reaching the electrode surface, thereby limiting the detection signal. An effective strategy to address this issue is to modify the electrode surface with a blocking agent, which acts as a barrier to prevent the adsorption/binding of irrelevant proteins. Blocking agents that have been used in biosensors include bovine serum albumin (BSA), casein, polyethylene glycol (PEG), and zwitterionic polymers [75]. However, a potential limitation of applying these materials on the electrode surface is increasing the electrical impedance of the sensor, which can diminish electron transfer and reduce the sensitivity of the sensor [76]. Therefore, alternative materials, such as conductive polymers or combinations of conductive and polymeric materials, have been developed to prevent nonspecific protein adsorption on electrochemical sensors for biomolecular detection in whole blood samples [77].

Another strategy to minimize nonspecific binding has been to block the sensor surface via steric hindrance effects. This can be accomplished using either nanomaterials or biomolecular complexes, such as biorecognition element-target complexes on the working electrode. One advantage of this approach is that the amount of steric hindrance can be modulated using different-sized nanomaterials and biorecognition elements as well as different surface densities of immobilized molecules, thus enabling close control of the molecules that can be transported to the electrode surface. A similar outcome can be achieved using nanoporous membranes, which can prevent cellular components and potentially interfering proteins in blood from reaching the sensing electrode. This technique can be tailored to remove biomolecules with varying molecular weights using different membrane materials and pore sizes. While based on different mechanisms, all of the aforementioned strategies minimize nonspecific binding on the sensor surface, which reduces the background signal, thus enhancing the analytical sensitivity.

### Electrode surface modification

While traditional blocking agents used in molecular assays have shown to be useful in minimizing nonspecific binding in electrochemical sensors, they can potentially impede the electron transfer between the reporter and working electrode, reducing the detection signal. To overcome this issue, combinations of materials, such as a blocking agent and a conductive polymer, have been used to prevent nonspecific binding while also facilitating electron transfer between the reporter and working electrode. This technique was employed by Zupančič et al. [78] who developed an electrochemical sensor containing a nanoporous composite coating to prevent biofouling while achieving high signal transduction. The composite coating consisted of cross-linked BSA and reduced graphene oxide (rGO) nanoflakes. The BSA-rGO coating enabled the sensor to maintain a steady current density for 60 min of incubation with a blood sample, demonstrating its ability to minimize nonspecific binding during long incubation periods. This sensor was used for measurements of procalcitonin (PCT), a biomarker for sepsis, which could be detected at concentrations as low as 24.7 pg/mL in 2×-diluted blood.

**Fig. 4:**
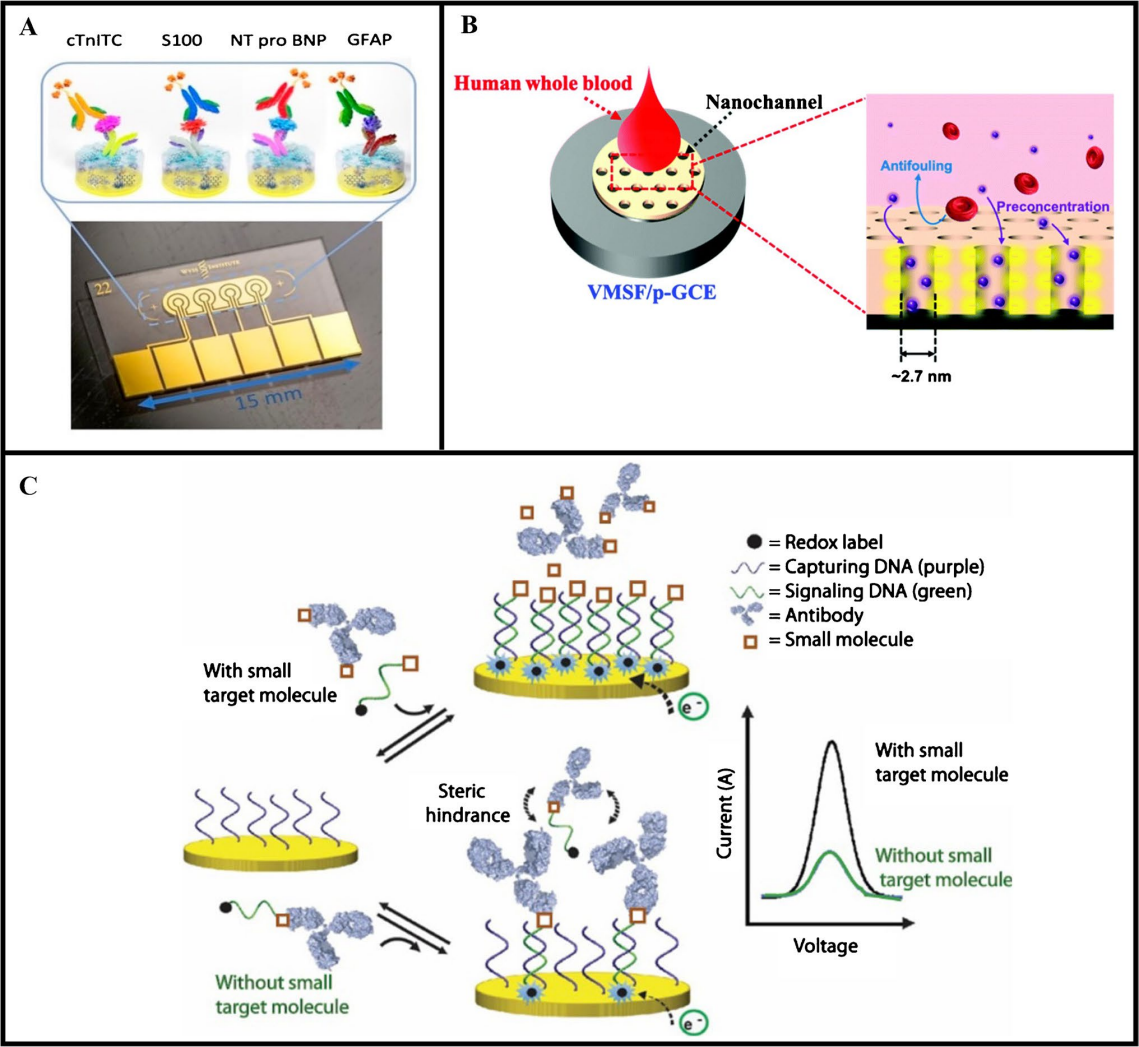
Strategies for reducing the background signal. **A** Multiplexed detection of cTnI-TC, S100, NT-proBNP, and GFAP on an electrochemical sensor employing a conductive nanoporous composite coating to prevent biofouling. Reprinted (adapted) with permission from [79]. **B** Molecular separation from a whole blood sample via passage through aligned nanochannels. Copyright 2021 American Chemical Society. Used with permission of Royal Society of Chemistry from [86]; permission conveyed through Copyright Clearance Center, Inc. **C** Blocking of target proteins from binding to the capture DNA-immobilized working electrode due to the steric hindrance effect. Reprinted (adapted) with permission from [90]. Copyright 2017 American Chemical Society

Timilsina et al. [79] studied the use of different nanoporous composites made of a combination of conductive nanomaterials and polymers to reduce the effects of biofouling on Au electrodes. The nanoporous composite consisted of glutaraldehyde cross-linked BSA combined with different conductive nanomaterials (Au nanowires, CNTs, or rGO nanoflakes), which allowed for the transport of the target biomolecules to the sensor surface while facilitating the flow of current through the composite. Using this sensing platform, various biomarkers including B-type natriuretic peptide (BNP), NT-proBNP, S100b, cardiac troponin I (cTnI), cardiac troponin noncovalent ternary complex (cTnI-TC), and glial fibrillary acidic protein (GFAP) could be detected at single pg/mL levels in 15 μL of undiluted whole blood (Fig. 4A). The use of nanoporous composites also enabled the development of a multiplexed electrochemical sensor with a similar layer consisting of denatured BSA cross-linked with pentaamine-functionalized graphene oxide cross-linked with glutaraldehyde [80]. This coating enabled the sensitive detection of several protein biomarkers, including GFAP and neurofilament light polypeptide (NF-L) in whole blood, with lower LODs of 2 pg/mL and 10 pg/mL, respectively. Timilsina et al. [81] further demonstrated that replacing rGO with pentaamine-functionalized graphene allowed the coating to be rapidly (< 1 min) deposited on Au electrodes. Coated sensors exhibited excellent long-term stability and could be stored for up to 20 weeks without a drop in sensor performance. Sensors were used for electrochemical measurements of cTnI-TC, GFAP, and S100b in whole blood, which could be detected at concentrations as low as 22 pg/mL, 2 pg/mL, and 13 pg/mL, respectively.

Due to their effectiveness in reducing nonspecific adsorption, zwitterionic materials, a class of materials that contain both cationic and anionic groups, have also been used to reduce the background signal in electrochemical sensors [82]. When combined with other materials to provide sites for molecular conjugation, zwitterionic species can effectively reduce the background signal as well as increase the analytical sensitivity. For example, Jiang et al. [76] reported a surface modification strategy using a mixed surface layer chemistry composed of zwitterionic species to minimize biofouling and other compounds to facilitate biomolecular conjugation. In this technique, a combination of phenyl phosphorylcholine and phenyl butyric acid was coated onto an indium tin oxide electrode, which enabled the capture antibody to be covalently attached to the electrode. The coated electrode maintained a constant charge transfer resistance in a redox probe solution containing human serum albumin for > 1 h, indicating that the coating was effective at preventing biofouling of the electrode surface. This sensor was then used for measurements of TNF-α, which could be detected at concentrations as low as 10 pg/mL in whole blood with similar accuracy (< 10% variability) as ELISA.

Heparin, an anticoagulant, has also been shown to be effective in reducing red blood cell and platelet adsorption on sensing electrodes [83]. Fang et al. [84] used this strategy to develop a MB-based electrochemical sensor for the detection of chromium(VI) in rabbit blood. The sensing electrodes were modified with heparin to prevent nonspecific binding and polyaniline, a conductive polymer, to improve the electrical conductivity of the coating. Tannin-modified MBs were then immobilized on the electrodes to oxidize chromium(VI) to chromium(III), which consequently generated an electrical current proportional to the concentration of chromium(VI). In addition to minimizing nonspecific binding through the use of the heparin-polyaniline electrode coating, the large surface area of the MBs resulted in a large oxidation reaction and amplified detection signal, enabling the detection of chromium(VI) at concentrations as low as 0.0452 ppm (0.87 µM) in 50×-diluted rabbit blood. Xu et al. [85] leveraged the antibiofouling effects of heparin and the benefits of nanomaterials to develop a biosensor for the detection of α-Fetoprotein (AFP) in blood. In this work, heparin (Hep) was combined with polyglutamic (PGA) and polpyrrole (PPy) to create conductive and biocompatible nanoparticles with antibiofouling properties. The Hep-PGA-Ppy nanoparticles were deposited on glassy carbon electrodes that were coated with an anti-AFP solution for specific molecular recognition. Measurements of AFP in rabbit blood revealed that this sensor exhibited a lower LOD of 0.099 ng/mL. Furthermore, AFP levels were accurately detected in five human blood samples with < 4% relative deviation compared with concentrations determined by a gold standard chemiluminescence method.

### Nanoporous membranes for molecular separation

Another strategy for minimizing nonspecific binding on the sensing electrode is using an engineered nanoporous membrane to separate out potentially interfering biomolecules, including proteins, from the blood sample. In contrast to commercially available plasma separation membranes which only filter cellular components from blood, nanoporous membranes can be fine-tuned for the removal of specific-sized and/or electrically charged molecules, in addition to blood cells and platelets. Therefore, the use of engineered nanoporous membranes enables the specific passage of the target biomarker(s) to the sensing electrode, preventing potentially interfering molecules from binding to the electrode. The use of a nanoporous membrane was shown to be effective by Wang et al. [86] who employed vertically ordered mesoporous silica films to create aligned nanochannels on electrochemically pretreated glassy carbon electrodes (Fig. 4B). These nanochannels functioned to filter the blood sample to prevent cells and large molecules from reaching the electrode surface, while also controlling the volume of plasma that was transported to the sensor, effectively concentrating the target biomarker at the electrode surface. The optimized device was used to detect doxorubicin in 50×-diluted whole blood, which exhibited two linear detection ranges of 0.5 nM to 2 μM and 2 to 20 μM, with sensitivities of 20.32 μA/μM and 2.696 μA/μM, respectively. The use of nanochannels showed a 19× improvement in sensitivity compared to electrochemically pretreated glassy carbon electrodes with no mesoporous film and a 65.5× improvement in sensitivity compared to standard electrodes.

While nanoporous membranes are typically used to filter large molecules, they have also been combined with other nanomaterials to prevent smaller electroactive molecules from reaching the electrode surface. Escosura-Muñiz and Merkoçi [87] developed an electrochemical immunosensor using an anodized aluminum oxide nanoporous membrane combined with AuNPs for the detection of cancer antigen 15–3 (CA15-3) in spiked whole blood. The nanoporous membrane was functionalized with anti-CA15-3 capture antibodies and immobilized onto the sensing electrode. Incubation of the target CA15-3 protein with AuNP-conjugated detection antibodies on the membrane resulted in the formation of sandwich immunocomplexes. The presence of the immunocomplexes on the membrane blocked the passage of other electroactive species through the membrane, reducing nonspecific binding on the electrode surface. The AuNPs were further modified using a silver enhancement treatment, which led to the growth of a silver shell around the AuNPs, increasing their size to perfectly fit the diameter of nanopores, further reducing flow through the membrane. Using this platform, CA15-3 could be detected at concentrations as low as 52 U/mL in spiked whole blood.

### Steric hindrance effects

The steric hindrance effect is a phenomenon that has been employed in electrochemical sensors to prevent nonspecific binding of the reporter on the working electrode. Rather than using an additional material, such as a nanoporous membrane, to filter out electroactive species from blood, steric hindrance uses the biorecognition element-target complexes themselves to block the surface of the electrode. At low biomarker concentrations, there are fewer complexes formed, which allows more reporter molecules to reach the electrode surface, resulting in a large electrochemical current. As more target molecules bind to the biorecognition elements immobilized on the electrode surface, there is less space for the reporter to bind to the electrode surface, diminishing the electrochemical signal. Steric hindrance also prevents other irrelevant molecules from reaching the electrode surface, which reduces the background signal, thereby increasing the analytical sensitivity. Mahshid et al. [88] reported an electrochemical sensor for the detection of macromolecules ranging in size from 50 to 150 kDa in bovine blood. In this work, steric hindrance was used to prevent a redox-labeled DNA reporter, which binds the target protein, from hybridizing with the capture DNA on the working electrode. When the target protein bound to the capture DNA, the steric hindrance from the target-DNA complex blocked the redox-labeled DNA from binding to the capture DNA, thus reducing the detection signal. Additionally, the use of highly specific DNA probes minimized nonspecific binding of blood components, resulting in a 10× increase in the detection signal compared with that of a standard DNA-based electrochemical sensor. Using this sensor, Fab fragment, streptavidin, anti-digoxigenin (anti-DIG), and anti-dinitrophenyl (anti-DNP) could be detected at concentrations as low as ~10 nM in undiluted bovine blood within 10 min. Sun et al. [89] also employed the steric hindrance effect to develop an electrochemical sensor for the detection of thrombin in whole blood. This sensor consisted of a self-assembled multilayer with the outermost layer containing a thrombin-binding aptamer (TBA). In the presence of thrombin, thrombin-TBA complexes were formed on the sensing electrode, thus preventing the transfer of electrons from the reporter to the electrode surface. As the concentration of thrombin increased, the peak electrochemical current decreased, indicating a decrease in the electron transfer rate. Using this sensor, measurements of thrombin were performed in whole blood, which could be detected at concentrations as low as 15.6 fM.

Mahshid et al. [90] further modified their DNA-based electrochemical sensor for the detection of small molecules, specifically digoxin, in bovine blood. In this work, the electrode surface was coated with capture DNA molecules and the detection signal was generated by the hybridization of a dually labeled methylene blue and digoxin DNA molecule with the capture DNA molecules. To initiate the measurement, anti-digoxin antibodies and the dually labeled signaling DNA were added to the sensor with the blood sample. In the presence of digoxin, the binding sites on the antibodies bound to digoxin, which limited the number of redox-labeled DNA reporters that could bind to the antibodies. In this case, the free redox-labeled DNA reporter hybridized with the capture DNA and produced a large detection signal. In the absence of digoxin, the digoxin binding sites on the antibodies bound to the redox-labeled DNA reporter, which then hybridized with the capture DNA. The steric hindrance due to the DNA-antibody complexes prevented further redox-labeled DNA reporter or other electroactive species from attaching to the electrode surface, thus reducing the detection signal (Fig. 4C). Using this sensor, digoxin could be detected in undiluted bovine blood from 1 to 30 nM within 20 min.

## Conclusions and future perspectives

Electrochemical sensors are powerful analytical tools capable of sensitive, quantitative measurements of molecular biomarkers in biofluids in a simple, rapid, and portable manner. However, the complex matrix of whole blood produces unique challenges for electrochemical sensing, such as nonspecific binding and adsorption of blood components on the sensor surface, which reduces the analytical sensitivity. Plasma separation via centrifugation can minimize the sample matrix effects associated with whole blood; however, this approach is tedious and requires expensive and bulky instrumentation, hindering its use for some diagnostic applications.

In this review, various strategies to improve the analytical performance of affinity-based electrochemical sensors for biomolecular detection in whole blood were presented, which are summarized in Table 1. One strategy that has been effective in minimizing the sample matrix effects associated with whole blood, while circumventing the need for bulky equipment or tedious sample handling, has been to separate plasma from the blood sample directly on chip. Other strategies include the use of conductive nanomaterials to increase the number of binding sites for the target biomarker and/or reporter, which has shown to be effective in amplifying the detection signal in whole blood samples. Furthermore, enhancement of the detection signal in whole blood has been achieved through the use of magnetic concentration and shape-changing aptamers, resulting in enhanced electron transfer during electrochemical reactions. A complementary strategy that has been demonstrated to prevent the nonspecific binding of potentially interfering components in blood on the sensor surface is the use of coatings comprised of a blocking agent and conducive polymer to reduce the background signal.

**Table 1:** Summary of affinity-based electrochemical sensors for biomolecular detection in whole blood

	Enhancement strategy	Target analyte(s)	Sample type*, dilution factor	Limit of detection**	Detection range***	Required blood volume	Ref
On-chip sample purification	Plasma separation	*Pf*HRP2	3×	-	100 ng/nL–100 μg/mL (dynamic)	10 μL	22
	Plasma separation	IL-6	3×	-	-	1–5 μL	31
	Plasma separation	SARS-CoV-2 anti-N antibody	Undiluted	5 ng/mL	-	10 μL	33
	Plasma separation	hr-HPV16-cDNA	Undiluted	0.48 μM	-	160 μL	34
	Microfluidic-based separation	Histamine	Undiluted	-	200–2000 ng/mL (dynamic)	10 μL	36
	Microfluidic-based separation	PSA, complement C3, CRP, plasminogen	Undiluted	-	-	10 μL	37
	Microfluidic-based separation	VEGF165	Undiluted	0.67 pg/mL	1 pg/mL–10 ng/mL	50 μL	39
	Electrokinetic-based separation	PSA	Undiluted	0.25 pg/mL	1 pg/mL–10 ng/mL	-	41
Signal enhancement	Conductive nanomaterials	HbA1c	Undiluted	1 ng/mL (buffer)	1–500 ng/mL	-	50
	Conductive nanomaterials	HbA1c	Undiluted	-	4.6–15.1%	-	51
	Conductive nanomaterials	H_2_O_2_	Undiluted	0.02 μM (buffer)	-	10 μL	52
	Conductive nanomaterials	Otolin-1, prestin	Undiluted	Otolin-1: 2 pM	-	10 μL	53
	Conductive nanomaterials	Hb, HbA1c	100×	HbA1c: 4.2 nM	Hb: 9.4–16.4 g/dL; HbA1c: 31.2–500 nM	2 μL	55
	Magnetic concentration	miR-21	2×	10 aM	10 aM–1 nM (dynamic)	100 μL	57
	Magnetic concentration	CRP	10–100×	1.5 ng/mL (buffer)	0.005–1.0 µg/mL	5 μL	58
	Magnetic concentration	NT-proBNP	Unspecified blood, unspecified	0.003 ng/mL (buffer)	-	-	59
	Magnetic concentration	*Pf*LDH	25×	8.5 ng/mL	6.25–100 ng/mL	-	60
	Magnetic concentration	Ox-LDL	Unspecified blood, 100×	9.8E−4 μg/mL	1E−2–10 μg/mL	-	61
	Magnetic concentration	miRNA-141, thrombin	Unspecified blood, undiluted	miRNA-141: 4.73 aM; thrombin: 29.60 aM	-	1 mL	62
	Magnetic concentration	*Pf*HRP2	5×	5.7 pg/mL	0–5000 pg/mL	16 μL	63
	Aptamer probes	Phenylalanine	1000×	-	0.1–10 μM (dynamic)	0.1 μL	66
	Aptamer probes	TNF-α	Undiluted	58 pM (10 ng/mL)	10–100 ng/mL	-	67
	Aptamer probes	VEGF	Undiluted	-	-	-	68
	Aptamer probes	VSL12	Bovine blood, undiluted	-	-	-	69
	Aptamer probes	Cocaine, doxorubicin	Bovine blood, undiluted	-	Cocaine: 60 μM–1 mM, 20 μM–2 mM (unspecified) Doxorubicin: 10–60 μM, 4–100 μM (unspecified)	-	70
	Aptamer probes	DNA, ATP	Undiluted	DNA: 300 fM (unspecified); ATP: 5 nM (unspecified)	-	-	71
	Aptamer probes	HbA1c, tHb	100–100,000×	HbA1c: 0.2 ng/mL (buffer); tHb: 0.34 ng/mL (buffer)	100 pg/mL–100 ng/mL	1 μL	73
	Aptamer probes	ctDNA	Unspecified, undiluted	1 pM (buffer)	0.1 pM–1 nM	-	74
Background signal reduction	Electrode surface modification	TNF-α	Undiluted	10 pg/mL	0.01–500 ng/mL	-	76
	Electrode surface modification	PCT	2×	24.7 pg/mL	0.07–2.49 ng/mL (dynamic)	-	78
	Electrode surface modification	GFAP, NF-L	Undiluted	GFAP: 2 pg/mL; NF-L: 10 pg/mL	0.01–10 ng/mL (unspecified)	15 µL	80
	Electrode surface modification	CtNI-TC, GFAP, S100b	Undiluted	CtNI-TC: 22 pg/mL; GFAP: 2 pg/mL; S100b: 13 pg/mL	0.01–10 ng/mL	15 μL	81
	Electrode surface modification	Cr(VI)	Rabbit blood, 50×	0.0452 ppm (0.87 μM)	10E−7–10E−4 ppm	-	84
	Electrode surface modification	AFP	Rabbit blood, unspecified	0.099 ng/mL	0.1–100 ng/mL	-	85
	Nanoporous membranes	Doxorubicin	Undiluted	0.2 nM (buffer)	0.5 nM–23 μM	-	86
	Nanoporous membranes	CA15-3	Undiluted	52 U/mL	60–240 U/mL	-	87
	Steric hindrance effect	Anti-DIG, anti-DNP	Bovine blood, undiluted	~10 nM	100 nM–1 μM (dynamic)	979 μL	88
	Steric hindrance effect	Thrombin	Undiluted	15.6 fM	1 pM–160 nM	-	89
	Steric hindrance effect	Digoxin	Bovine blood, undiluted	1 nM	10 nM–1 μM (dynamic)	100 μL	90

^*^Measurements were performed in human blood unless noted otherwise

^**^LOD was obtained in blood unless noted otherwise

^***^Linear detection range is presented unless noted otherwise

Generally, each of these strategies addresses one specific obstacle associated with biomolecular detection in whole blood. Therefore, future research in this space should focus on using a combination of strategies, which can provide further improvements in sensor performance. For example, incorporating conductive nanomaterials, which have been shown to amplify the detection signal, with filtration membranes and/or coating the electrode surface with blocking agents could further minimize nonspecific binding. Such combinatorial techniques could also include incorporating nanoparticles into sample purification, surface modification, and electrokinetics-based separation techniques to improve the sensor performance in regard to the analytical sensitivity, selectivity, and detection time. Another future area of research could capitalize on the recent advances in microfluidics to enable more complex sample processing in an automated format with minimal user involvement. Additionally, to expand on the manufacturability for future commercialization, integrating affinity-based electrochemical sensors onto low-cost, disposable materials, such as paper and textile, would be advantageous for reducing device costs. All of the aforementioned strategies could further expand the capabilities of existing biosensors, enabling faster and more sensitive detection of molecular biomarkers in whole blood, thus allowing for more accessible and accurate diagnostics in clinical and POC settings.
